# Supplementary material for: Bacterial degradation of ctenophore Mnemiopsis leidyi organic matter
Source: mSystems. 2024 Jan 23;9(2):e01264-23. doi: 10.1128/msystems.01264-23 (PMC10878102; doi:10.1128/msystems.01264-23)
Supplement: Supplemental material — Supplemental figures and table legends. [file msystems.01264-23-s0001.docx]

Supplementary Material

**Bacterial degradation of ctenophore *Mnemiopsis leidyi* organic matter**

Eduard Fadeev, Jennifer H Hennenfeind, Chie Amano, Zihao Zhao, Katja Klun, Gerhard J Herndl, Tinkara Tinta

## Supplementary figures


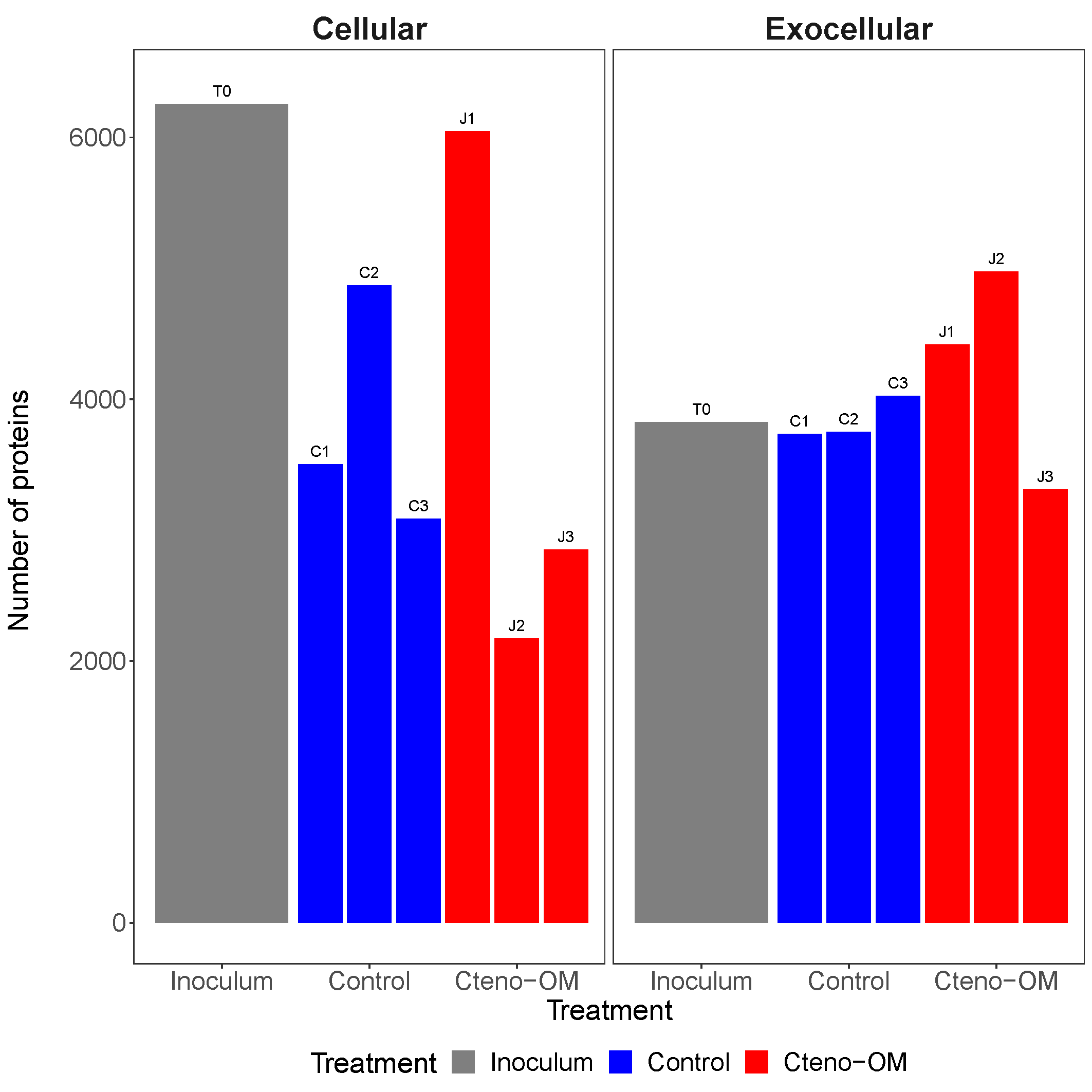


**Figure S1 -** Total number of bacteria-associated cellular (left) and extracellular (right) proteins in each microcosm and in the seawater inoculum.


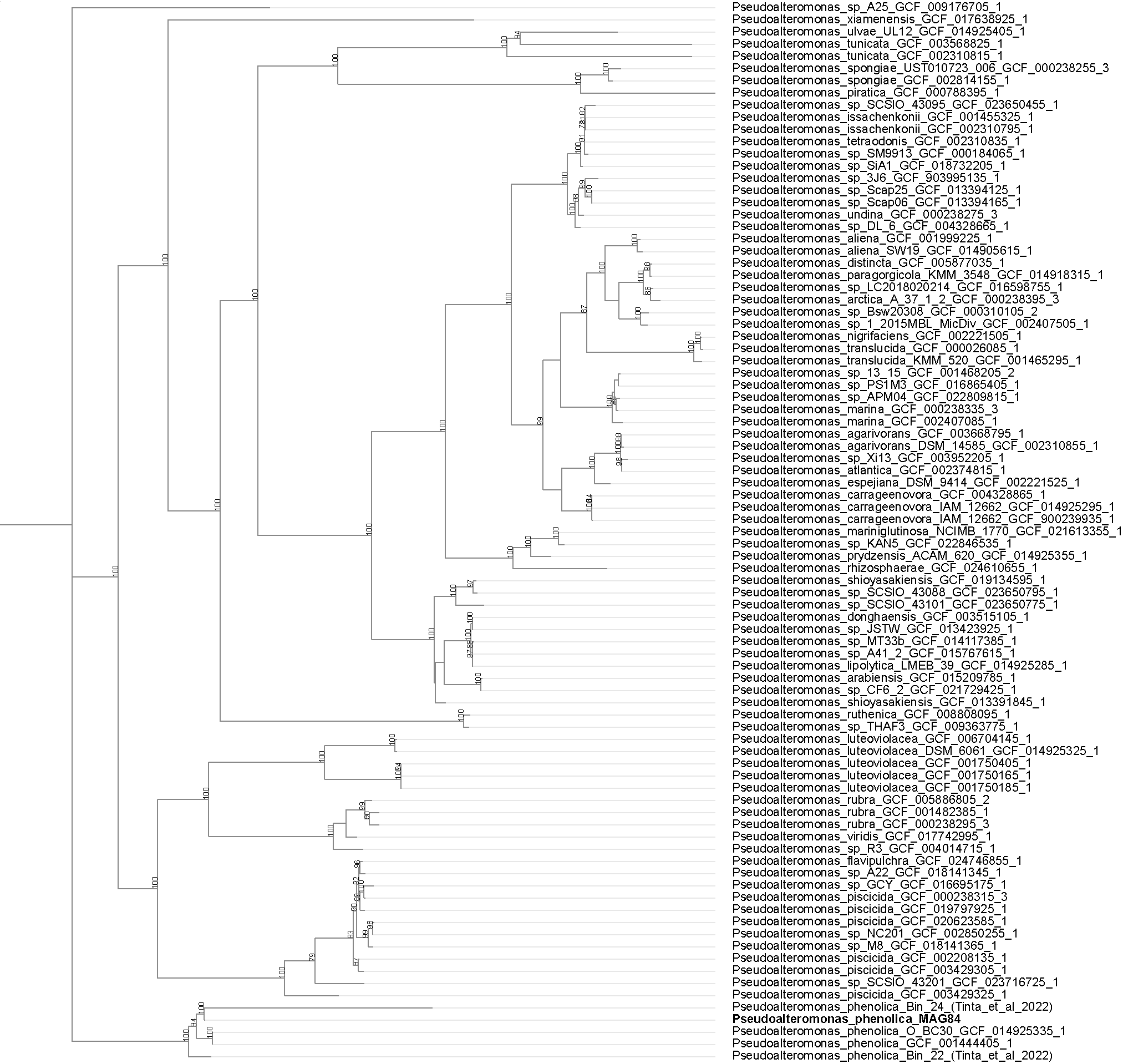


**Figure S2** – Phylogenetic tree of all complete Pseudoalteromonas genomes, and the reconstructed P. phenolica MAGs from this study (MAG84) and from Tinta et al. (2022) (Bin_24).


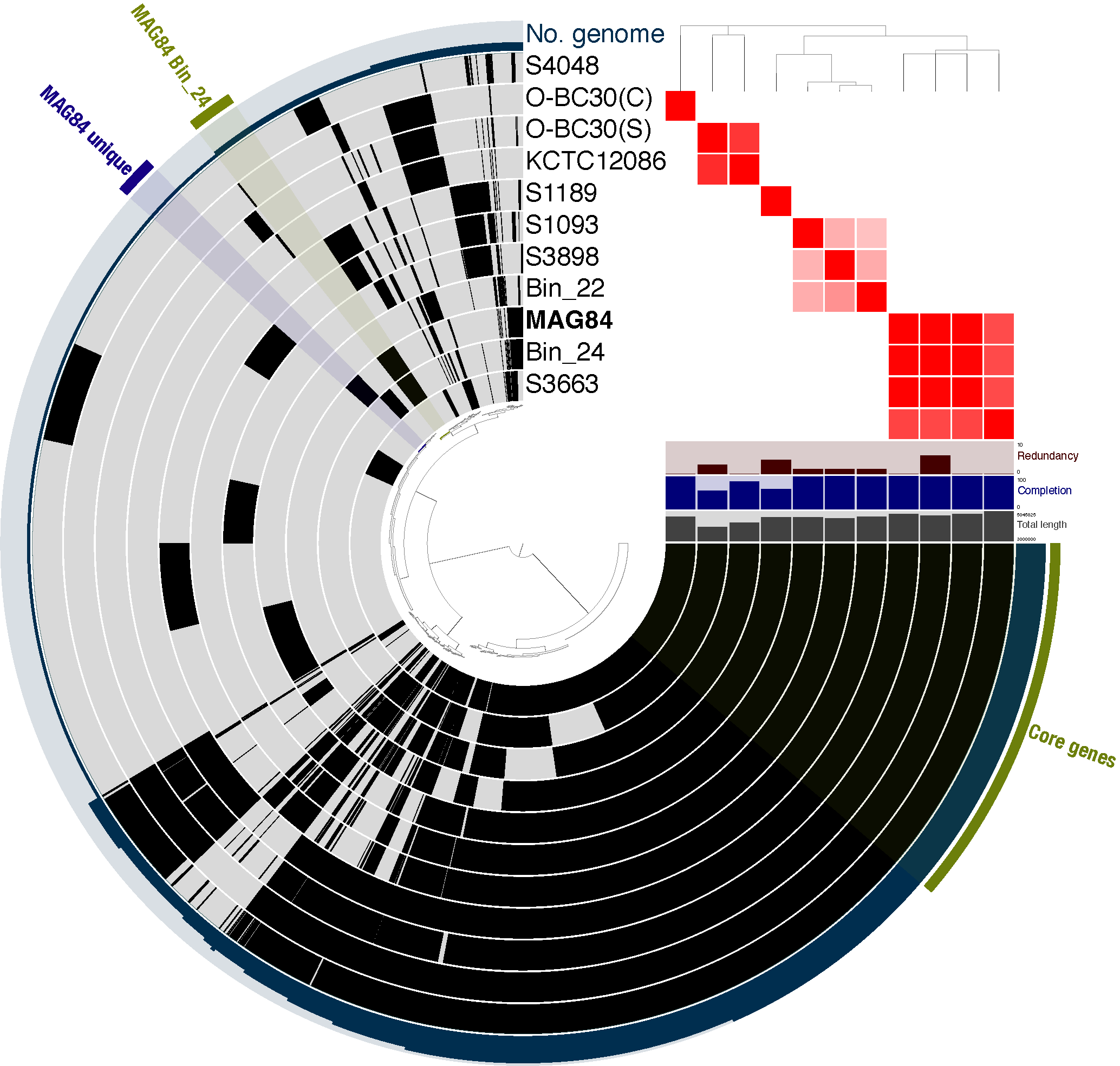


**Figure S3 -** Pangenome analysis of Pseudoalteromonas phenolica. The heatmap represents average nucleotide identity (95-100%), ordered according to phylogenetic tree. ‘MAG84 unique’ represents the genes found only in the P. phenolica MAG84, and “MAG84 Bin_24” represents the genes found only in the two MAGs but not in other genomes.

## Supplementary tables legends

**Table S1** – Protein results table from Proteome Discoverer.

**Table S2** – List of significantly enriched proteins and their annotations in Cteno-OM and Cotrol microscosms.

**Table S3** – Unique and shared genes between the P. phenolica MAGs.

**Table S4** – Taxa-specific oligonucleotide probes used for Fluorescence in situ hybridization (FISH).

**Table S5** – NCBI RefSeq accession numbers of P. phenolica used for the pangenome analysis.
